# Drug-Induced Modulation of T Lymphocytes as a Potential Mechanism of Susceptibility to Infections in Patients with Multiple Myeloma During Bortezomib Therapy

**DOI:** 10.1007/s12013-014-0224-x

**Published:** 2014-10-25

**Authors:** Juan Li, Ying Li, Beihui Huang, Dong Zheng, Mei Chen, Zhenhai Zhou

**Affiliations:** Department of Hematology, First Affiliated Hospital of Sun Yat-sen University, Guangzhou, 510080 Guangdong Province China

**Keywords:** Infection, Multiple myeloma, Chemotherapy, Bortezomib, T lymphocyte subsets

## Abstract

Bortezomib is effective in the therapy of multiple myeloma (MM), but causes infections that are different from those associated with conventional chemotherapy. It is important to identify the risk factors that facilitate infections associated with bortezomib therapy. In the present report, we sought to (1) define the features of the infections associated with this therapy and (2) identify the immune mechanisms responsible for the observed susceptibility to these infections. We first retrospectively analyzed the clinical data of 143 patients who had received bortezomib therapy for MM. We then prospectively assessed the modulation of T lymphocyte status during this therapy, and evaluated potential relationships between infections and T lymphocyte changes. The infection rates peaked during the first cycle of bortezomib therapy (47.6 %) in patients with MM (*p* < 0.05 vs. subsequent cycles). Bortezomib therapy was associated with higher incidence rates of viral and fungal infections (15.8 %, *p* < 0.05 vs. conventional chemotherapy). In addition, patients with the IgG immunophenotype showed higher bacterial and viral infection rates (respectively, *p* = 0.008 and 0.009). The T lymphocyte numbers significantly decreased after bortezomib therapy (*p* < 0.05), and the same was true for the Th1/Th2 ratio (*p* < 0.01). Patients with MM who have decreased lymphocyte counts, while on bortezomib therapy are more likely to develop bacterial or viral infections. In addition, an imbalance in T lymphocyte subsets is also associated with bacterial or viral infections in these patients.

## Introduction

Introduction of novel drugs and hematopoietic stem cell transplantation increased the remission rates and prolonged the survival of patients with multiple myeloma (MM) [1, 2]. However, infections still remain one of the major causes of death in these patients [3]. Studies suggest that it is both the tumor and suppression of immune function by chemotherapy drugs that increase the risk of infections in patients with MM [4]. Clinically, these infections typically feature atypical symptoms, rapid progression, and the presence of multiple pathogens [3, 4]. Advanced age of patients with MM and the frequently occurring multiple organ dysfunction complicate antimicrobial therapy [5]. Therefore, early prevention and diagnosis of infections are crucial for effective management of MM.

Bortezomib, a proteasome inhibitor, is currently the first-line medication in the treatment of MM. The drug is characterized by rapid action, high efficacy, and low bone marrow toxicity [2]. Bortezomib does not affect the incidence of infections [6]. However, when they occur, these infections are different from those associated with conventional chemotherapy. Specifically, these infections are caused by the less commonly occurring pathogens, such as herpes zoster, and neutropenia is not the only causal factor predisposing to these infections [7]. It is, therefore, important to identify other risk factors that facilitate infections associated with bortezomib therapy. In the present report, we sought to address the following two objectives: (1) to define the features of the infections associated with this therapy and (2) to identify the immune mechanisms responsible for the observed susceptibility to these infections. To this end, we retrospectively assessed clinical features of infections in 143 patients with MM who underwent the therapy with bortezomib, and prospectively examined in 30 patients the changes in T lymphocyte subsets during this therapy.

## Materials and Methods

### Patients

The international diagnostic and staging criteria for MM [8, 9] were utilized in our study. The first objective of the study was to retrospectively assess infections in 143 patients with MM who received bortezomib therapy in the Department of Hematology, First Affiliated Hospital of Sun Yat-sen University, from January 2006 to May 2012. We collected information on conditions of these patients, changes in blood cell counts, and other particularities of the infections. These data were compared with those of 124 patients with MM who underwent conventional chemotherapy (i.e., without bortezomib) during the same period of time. The data from both groups of patients are presented in the Table 1.

**Table 1:** Demographic and clinical data of study patients

Parameter	Bortezomib therapy (*n* = 143)	Conventional chemotherapy (*n* = 124)	*p*
Gender (M/F)	96/47	59/65	0.001
Median age (years, range)	56 (27–78)	62 (34–78)	<0.001
D-S staging (2/3)	11/132	17/107	0.109
ISS staging (1/2/3)	40/30/73	47/20/57	0.202
Immunophenotyping			
IgG/IgA/light chain/IgD	68/26/39/10	81/27/12/4	<0.001

In the prospective part of the study, we assessed the T lymphocyte status in 30 patients who were treated with bortezomib and 12 patients treated conventionally. The latter patients served as controls. Patients from both groups were also part of the above patient cohort.

### Treatment

Patients treated with bortezomib received VD regimen (bortezomib 1.3 mg/m^2^ on days 1, 4, 8, and 11; dexamethasone 20 mg/day from day 1 to day 4) and PAD regimen (bortezomib 1.3 mg/m^2^ on days 1, 4, 8, and 11; liposomal doxorubicin 40 mg/m^2^ on day 4; dexamethasone 20 mg/day from day 1 to day 4). A total of 545 cycles of bortezomib chemotherapy were administered to 143 patients with MM.

Conventional chemotherapy included VADM regimen (vincristine 0.5 mg/day from day 1 to day 4; pirarubicin 8 mg/m^2^ from day 1 to day 4; dexamethasone 20 mg/day from day 1 to day 4; melphalan 9 mg/m^2^ from day 1 to day 4), DVD regimen (vincristine 1.4 mg/m^2^ on day 1; liposomal doxorubicin 40 mg/m^2^ from day 1 to day 4; dexamethasone 20 mg/day from day 1 to day 4), and VAD regimen (vincristine 0.5 mg/day from day 1 to day 4; pirarubicin 8 mg/m^2^ from day 1 to day 4; dexamethasone 20 mg/d from day 1 to day 4). A total of 460 cycles of conventional chemotherapy were administered to 124 patients with MM.

The study was approved by the Human Ethics Committee of our Hospital, and informed consents were obtained from all the subjects.

### Definition and Classification of Infections

Infectious pathogens assessed in this study included bacteria, viruses, and fungi. We defined the time from the onset of infection-related symptoms/signs and radiographic/laboratory abnormalities until complete remission of the symptoms with negative radiographic and laboratory findings after specific treatment as one infection incident. The number of infection incidents per treatment cycle was calculated in each group.

The infections were classified based on their pathogenic nature into bacterial, viral, and fungal infections. Bacterial infections included those caused by Gram-positive bacteria, such as *Staphylococcus aureus* and *Streptococcus pneumoniae*, and those by Gram-negative bacteria, such as *Escherichia coli* and *Klebsiella pneumoniae* [10]. Viral infections included the ones caused by varicella-zoster viruses, hepatitis B viruses, and herpes simplex viruses [11–13]. Fungal infections included infections with *Aspergillus* and *Candida* [14, 15].

### T Lymphocyte Isolation and Culture

Six millilitre of fasting venous blood was collected from the cubital vein in early mornings of each day before the initial, second, third, and fifth cycles of chemotherapy. The blood was anticoagulated with heparin and EDTA, and used for complete blood cell count, analysis of T lymphocyte subsets, serum immunofixation electrophoresis, urinary Bence Jones protein testing, serum immunoglobulin analysis, and β2-microglobulin detection. Peripheral blood mononuclear cells were isolated using the human lymphocyte separation medium (ICN Biomedical Inc., Aurora, USA), washed with PBS (HyClone Laboratories, Logan, USA), and cultured in RPMI 1640 medium containing 10 % fetal calf serum (Life Technologies; Cergy, Pontoise, France).

### Cell Stimulation

A 3 ml aliquot of blood mononuclear cells was added to a culture dish and stimulated with 3 μl of 50 μg/l PMA, 3 μl of 1 μmol/l ionomycin (both from Sigma-Aldrich; St. Louis, USA), and 2 μl of 2 μmol/l monensin (BD Biosciences; San Jose, USA). Subsequently, the cells were incubated at 37 °C for 5 h. After stimulation, the cells were stained with antibodies for 30 min. The antibodies included Multitest 6-color TBNK, Th cell detection kit, CD69 APC (all from BD Biosciences), and Treg cell kit (eBioscience; San Diego, USA). The cells were washed and resuspended in PBS, and then analyzed by flow cytometry (BD FASCanto system) for expressions of CD69. The expression level of higher than 90 % was defined as cell activation.

### Flow Cytometry of Peripheral Blood T Cell Subsets

Unstimulated samples were stained with CD45-PerCP-Cy5.5, CD3-FITC, CD4-PE-Cy7, and CD8-APC-Cy7 monoclonal antibodies (all from BD Biosciences), and the ratio of peripheral Th/CTL cells was quantified. Further, unstimulated samples were stained with CD4-FITC and CD25-APC (eBioscience). Then, the cells were permeabilized using permeabilization buffer (eBioscience) and subsequently stained intracellularly with FOXP3-PE (eBioscience) to quantify the ratio of peripheral Treg cells.

Stimulated samples were stained using CD4-APC (BD Biosciences). The cells were permeabilized using the BD Cytofix/Cytoperm buffer (BD Bioscience) and subsequently stained intracellularly with IFN-γ-FITC, IL-4-PE, and Th-17-PE antibodies to quantify the ratio of peripheral Th1, Th2, and Th17 cells.

**Fig. 1:**
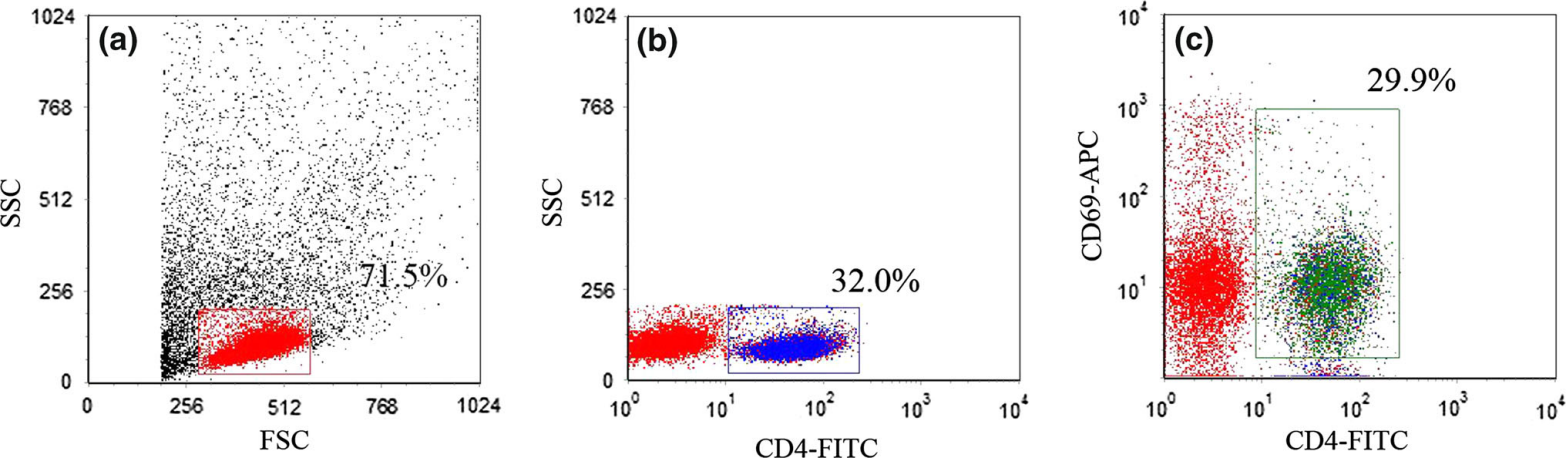
Flow cytometry analysis of CD69 expression in stimulated lymphocytes. **a** Gate set on peripheral blood lymphocytes; **b** gate set on CD4^+^ T lymphocytes; and **c** gate set on CD69^+^ T lymphocytes. *Numbers* represent respective percentages

**Fig. 2:**
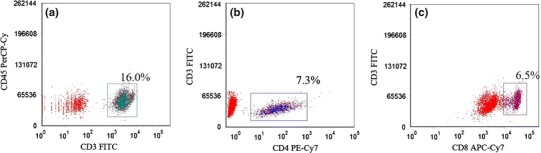
Flow cytometry analysis of lymphocyte subsets. **a** Gate set on CD45^+^ CD3^+^ T lymphocytes; **b** gate set on CD3^+^ CD4^+^ Th cells; and **c** gate set on CD3^+^ CD8^+^ CTL cells. *Numbers* represent respective percentages

**Fig. 3:**
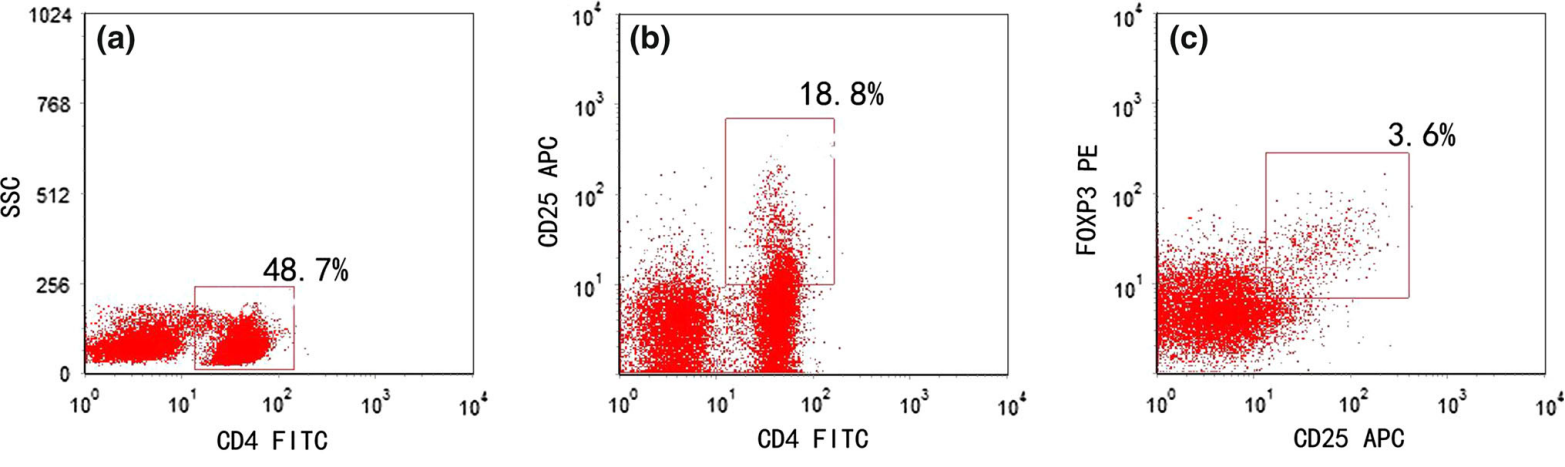
Flow cytometry analysis of Treg cells. **a** Gate set on CD4^+^ Th cells; **b** gate set on CD4^+^ CD25^+^ Th cells; and **c** gate set on CD4^+^ CD25^+^ FOXP3^+^ Treg cells. *Numbers* represent respective percentages

**Fig. 4:**
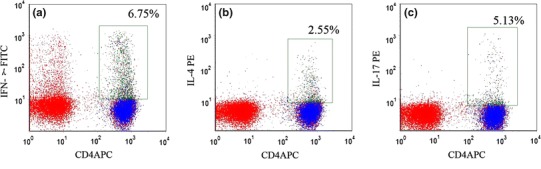
Flow cytometry analysis of Th1, Th2, and Th17 cells. **a** Gate set on CD4^+^ IFN-γ^+^ Th1 lymphocytes; **b** gate set on CD4^+^ IL-4^+^ Th2 lymphocytes; and **c** gate set on CD4^+^ IL-17^+^ Th17 lymphocytes. *Numbers* represent respective percentages

The cytometric data were analyzed using BD FASCanto system (BD Biosciences). The analysis procedure was done as shown in Figs. 1, 2, 3, and 4.

### Statistical Analysis

The statistical analysis was performed with SPSS 17.0 (SPSS Inc.; Tokyo, Japan). Categorical variables were analyzed using the Chi-square test, not normally distributed quantitative variables using the Wilcoxon test, and normally distributed data by *t* test. A Spearman correlation analysis was conducted to assess the correlation between categorical variables. A *p* value of <0.05 was defined as statistically significant.

## Results

### Infections in Patients Receiving Different Chemotherapy Regimens

**Fig. 5:**
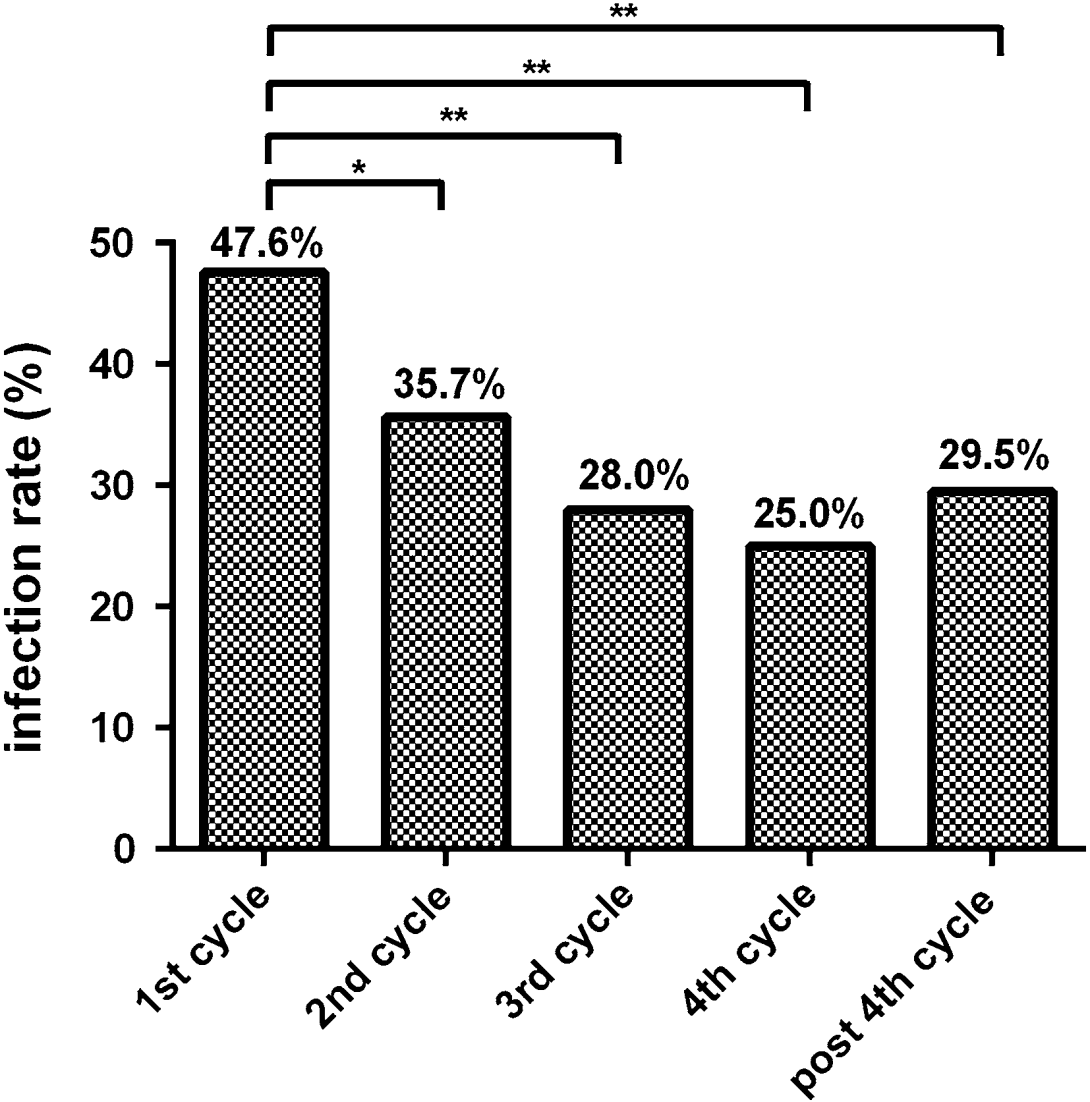
Infection rates during treatment cycles in patients receiving bortezomib therapy or conventional chemotherapy. * *p* < 0.05; ** *p* < 0.01

The overall infection rate was slightly higher in patients who received bortezomib compared with those on conventional chemotherapy (respectively, 34.9 vs. 30.7 %, *p* = 0.079). Specifically, the overall infection rate was significantly higher in the bortezomib group during the first cycle of therapy (*p* < 0.05 vs. the subsequent cycles; Fig. 5), while there were no significant differences during subsequent cycles (Fig. 5).

**Fig. 6:**
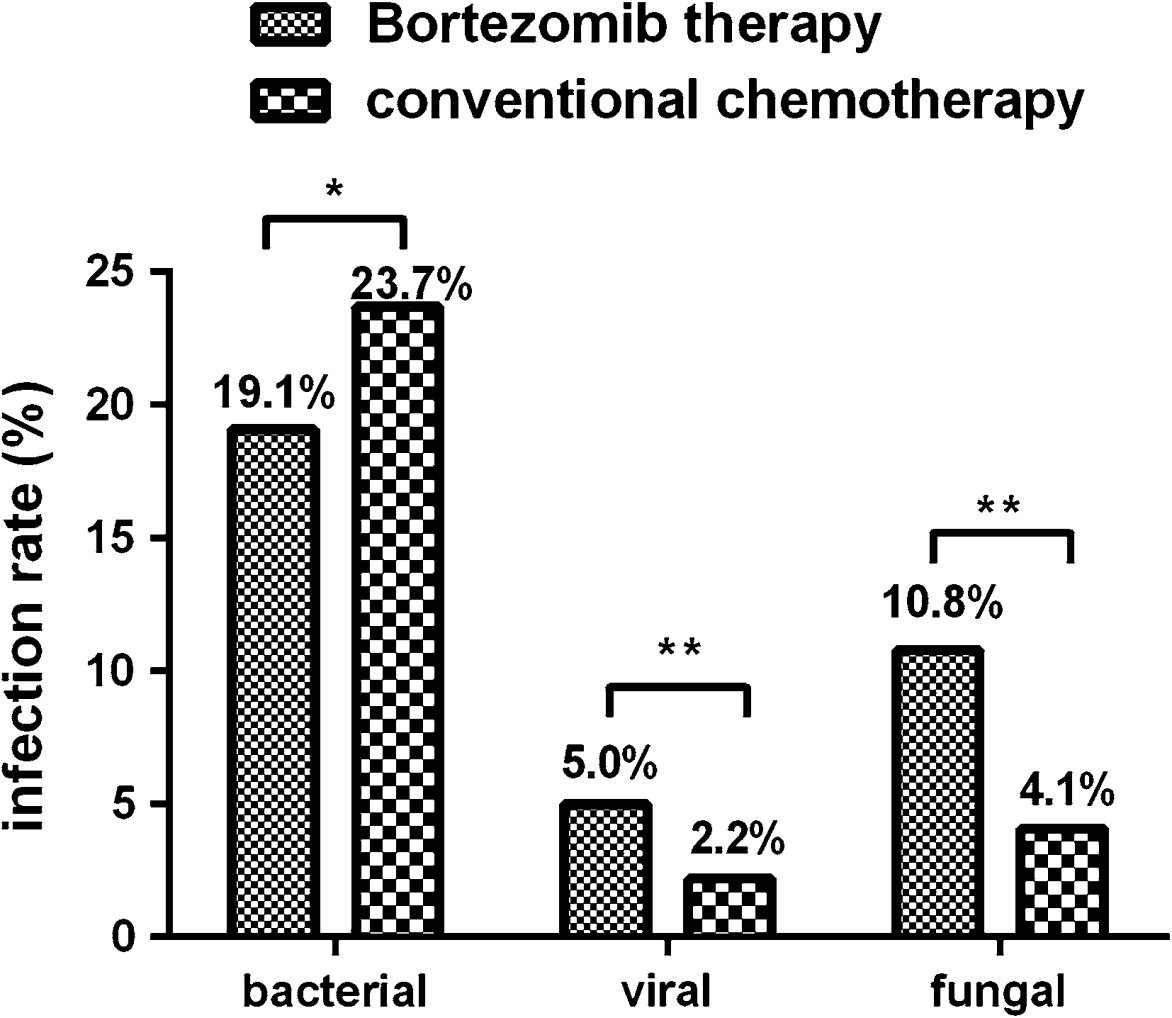
Infections with different pathogens in patients receiving bortezomib therapy or conventional chemotherapy. * *p* < 0.05; ** *p* < 0.01

**Fig. 7:**
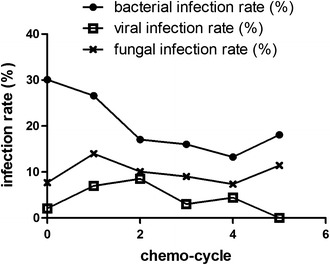
Kinetics of rates of infection with different pathogens in the bortezomib group

Further, the use of bortezomib was associated with higher incidence rates of viral and fungal infections (*p* < 0.05 vs. conventional chemotherapy). Conversely, the rates of bacterial infections were lower in the bortezomib group (*p* = 0.038 vs. conventional chemotherapy; Fig. 6). In addition, the first two cycles of bortezomib therapy were associated with significantly higher incidence rates of bacterial and viral infections, compared with the subsequent cycles (respectively, 28.3 vs. 17.5 %, *p* = 0.032; 8.4 vs. 2.2 %, *p* = 0.002; Fig. 7).

Following bortezomib therapy, patients with the IgG immunophenotype had a significantly higher infection rate compared with those with non-IgG immunophenotypes (42.2 vs. 28.6 %, *p* < 0.001). Further analysis revealed that this was due to higher bacterial and viral infection rates (respectively, 23.4 vs. 15.1 %, *p* = 0.008; 7.3 vs. 2.9 %, *p* = 0.009), while there was no difference in fungal infection rate between those with or without IgG immunophenotypes (11.5 vs. 10.4 %).

### Relationship Between Lymphocyte Counts and Infections

After the therapy with bortezomib, decreased lymphocyte counts (<1.0 × 10^9^/l) were observed in 23.6 % of patients with MM. Also, these patients showed a higher infection rate compared with patients with normal lymphocyte counts (38.9 vs. 30.8 %, *p* = 0.007). Furthermore, patients with decreased lymphocytes were more likely to have higher infection rates if they were treated with bortezomib (44.7 vs. 31.1 % in those with normal lymphocytes; *p* = 0.002), whereas no such significant difference was observed in patients who received conventional chemotherapy (32.6 vs. 30.4 %). A lymphocyte count of <1.0 × 10^9^/l weakly correlated with the occurrence of infection following chemotherapy in the bortezomib group (*R* = 0.127, *p* = 0.044).

In the bortezomib group, patients with decreased lymphocyte counts had a significantly higher rate of viral infections compared with those with normal lymphocyte counts (8.7 vs. 3.5 %, *p* = 0.007). However, this was not true for bacterial and fungal infections. A lymphocyte count of <1.0 × 10^9^/l weakly correlated with the occurrence of viral infections following bortezomib therapy (*R* = 0.105, *p* = 0.048).

### Effects of Bortezomib Regimen on T Lymphocytes

**Fig. 8:**
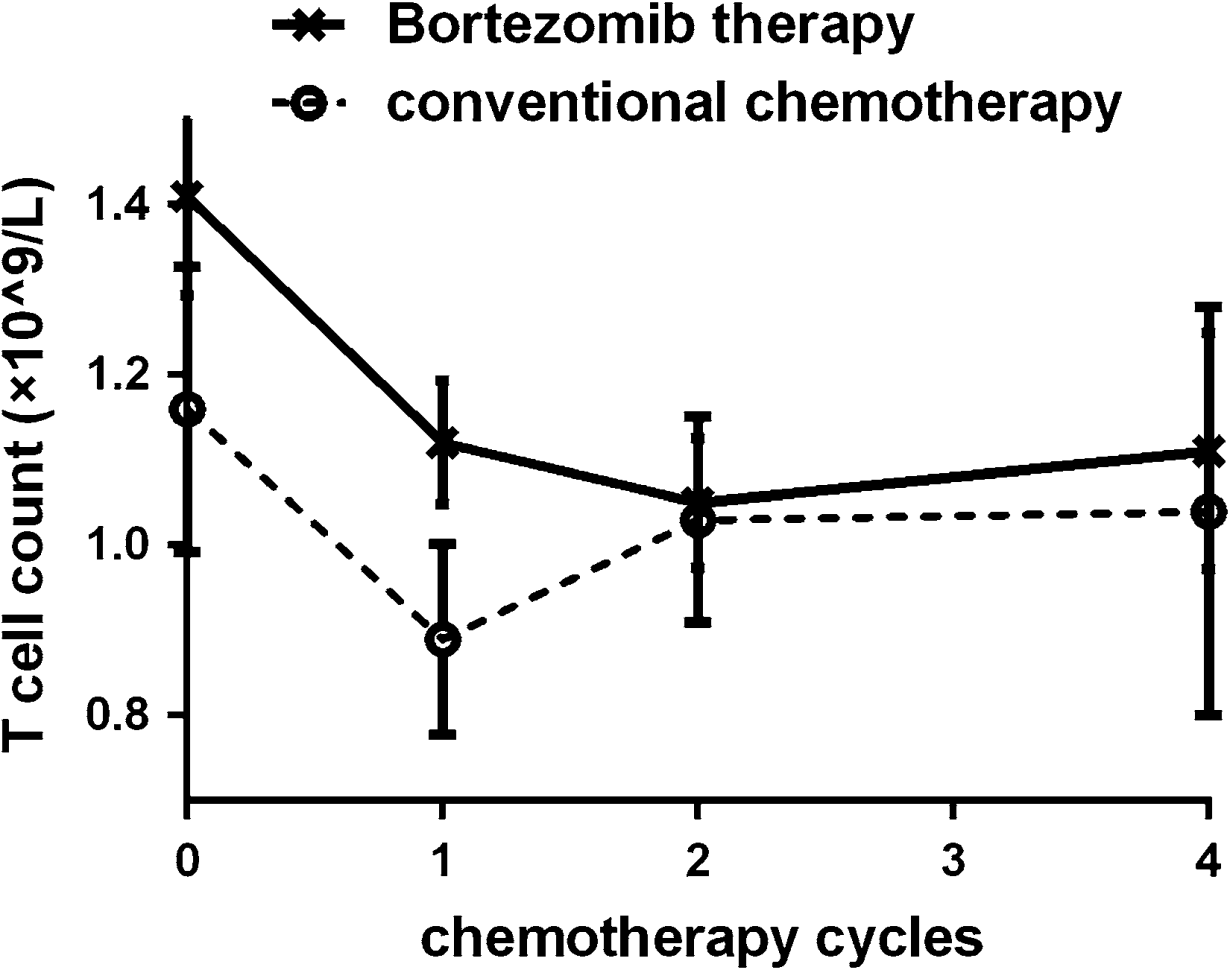
Kinetics of changes in T lymphocytes in patients receiving bortezomib therapy or conventional chemotherapy

Bortezomib therapy decreased T lymphocyte counts following the first, second, and forth cycles (*p* < 0.05 vs. baseline levels; Fig. 8). By contrast, in the conventional chemotherapy group, these decreased counts were only observed during the first cycle (*p* = 0.039; Fig. 8).

**Fig. 9:**
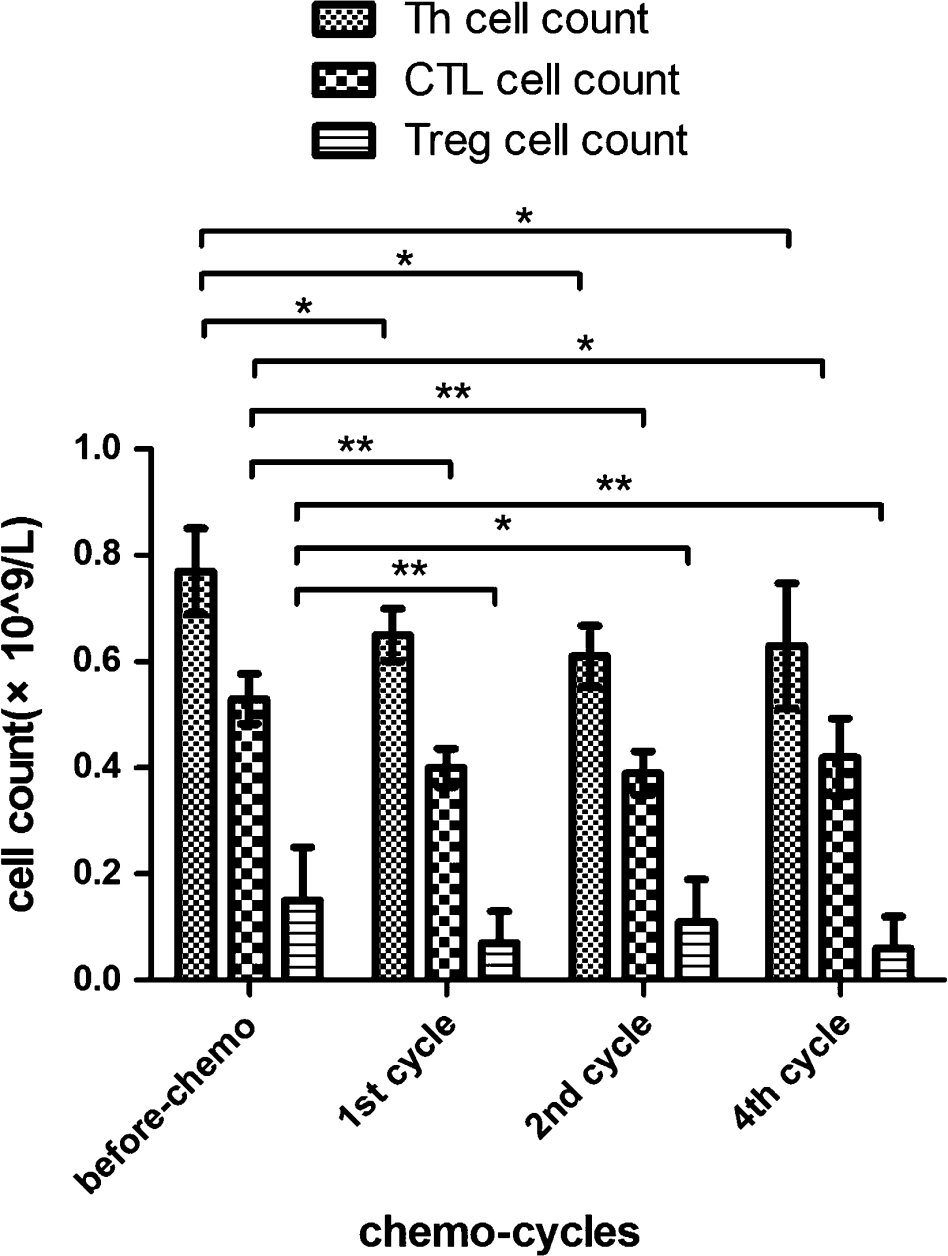
Changes in Th and CTL lymphocytes following the bortezomib therapy. * *p* < 0.05; ** *p* < 0.01

**Fig. 10:**
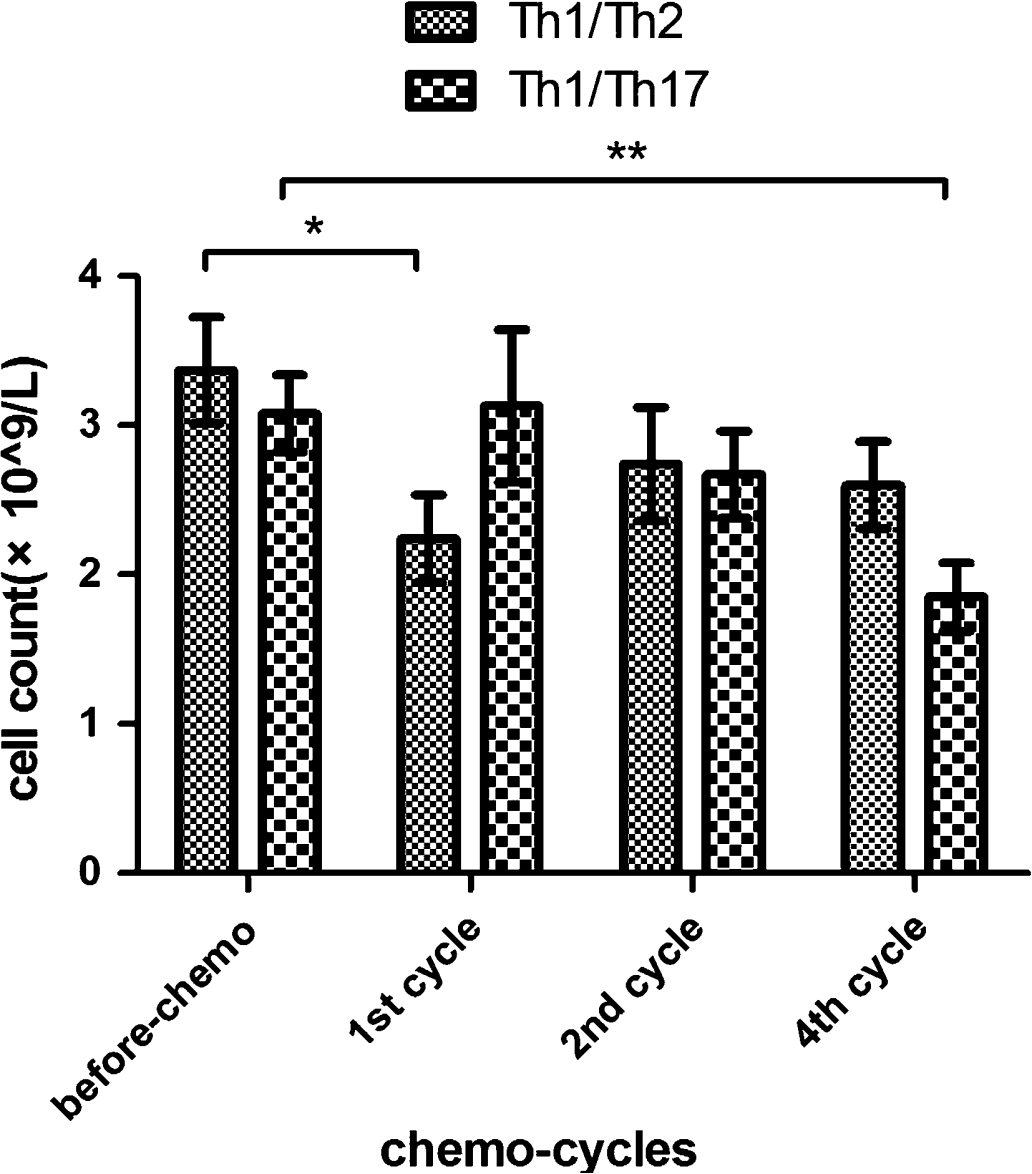
Changes in Th1/Th2 and Th1/Th17 ratios following the bortezomib therapy. * *p* < 0.05; ** *p* < 0.01

A further analysis of T lymphocyte subsets showed decreased counts of the Th, CTL, and Treg subsets in the bortezomib group during the first, second, and forth cycles,compared with the baseline (*p* < 0.05; Fig. 9). Based on the secreted cytokines and functions, Th cells were divided into the Th1, Th2, and Th17 subsets. Following the dynamic monitoring and analysis, the lowest Th1/Th2 ratio and Treg counts were observed after the first cycle of bortezomib therapy (*p* < 0.01), while the lowest Th1/Th17 ratio was found after the forth cycle (*p* = 0.007; Fig. 10).

In the bortezomib group, patients with bacterial infections had a significantly lower Th1/Th2 ratio (1.94 vs. 1.97, *p* = 0.047 vs. patients without bacterial infections). In addition, the Th1/Th2 ratio after therapy with bortezomib moderately correlated with the occurrence of bacterial infection (*R* = 0.574, *p* = 0.047). By contrast, patients treated with conventional chemotherapy did not show the association observed above (2.09 vs. 2.53 in patients with and without bacterial infections; *p* = 0.473).

A lower Th1/Th2 ratio was also frequently found in patients who received bortezomib and experienced viral infections (1.56 vs. 1.93, *p* = 0.026 vs. patients without viral infections) (Table 2). Similar to bacterial infections, the Th1/Th2 ratio after bortezomib chemotherapy moderately correlated with the incidence rates of viral infections (*R* = 0.463, *p* = 0.042) (Table 2). Since no viral infections occurred in 12 patients on conventional chemotherapy, no analysis of correlation between viral infections and Th1/Th2 ratio was carried out (Table 2).

**Table 2:** The Th1/Th2 ratio in infected and non-infected study patients

	Bacterial infection		Viral infection	
	Yes	No	Yes	No
Bortezomib therapy	1.94 (0.06–3.81)	1.97 (0.21–6.54)*	1.56 (0.11–2.28)	1.93 (0.06–3.81)*
Conventional chemotherapy	2.09 (0.46–5.79)	2.53 (0.83–5.86)	0 (0–0)	2.57 (1.37–3.21)

* *p* < 0.05 between infected and non-infected patients

## Discussion

The use of hematopoietic stem cell transplantation and new pharmacological drugs, such as bortezomib, thalidomide and lenalidomide, helped to achieve higher remission rates and to prolong survival in patients with MM [2]. However, the rates of infections associated with the therapy did not diminish, although the pattern of these infections changed [4].

As observed in our study, patients receiving bortezomib therapy experienced most infections following the first cycle of chemotherapy, with a gradual decrease in the subsequent cycles. Further, patients who received this therapy had infections with different pathogens compared with patients on conventional chemotherapy.

The unique clinical features associated with infections in the bortezomib group may be caused by pharmacological mechanisms of this agent. As a proteasome inhibitor, bortezomib primarily blocks the NF-κB pathway, inhibiting cell proliferation and differentiation. In addition to blocking the proliferation of tumor cells, it also inhibits normal T cells, NK cells, DC cells, and other immune cells [15–18]. Animal studies demonstrated that bortezomib can diminish T cell responses against viruses, reduce Th1-type immune responses, and promote viral proliferation [15–18]. People with functional defects in T lymphocytes are known to have higher incidences of viral and fungal infections [19]. In this regard, our study also revealed higher viral and fungal infection rates in patients with MM on bortezomib therapy. Therefore, we hypothesized that bortezomib-related clinical infections in these patients are associated with declined immune function of the lymphocytes.

Our studies confirmed the hypothesis. Specifically, decrease in lymphocyte numbers correlated with the incidence of post-therapy infections in the bortezomib group, which was different from patients receiving conventional chemotherapy. This was consistent with previously published findings [6]. Also, it was observed by others [20] that bortezomib inhibits T lymphocyte subsets in treated patients and disturbs the balance among lymphocyte subsets. Our analysis of the changes in lymphocyte subsets after therapy with bortezomib showed an overlap between the peak of inhibition of T cell subsets and peak occurrence of infections. Subsequently, we demonstrated that the Th1/Th2 ratio after bortezomib therapy correlated with the incidence of viral infections.

Thereby, we demonstrate that infections occurring in patients with MM during bortezomib therapy are associated with T lymphocyte functional defects. Therefore, patients with lymphocytopenia and Th1/Th2 imbalance after this therapy should be regarded as being at high risk for infections and should be managed with strict infection control measures. In these patients, preventive measures to improve their neutrophil counts and humoral immune functions are not sufficient. Since the infections following bortezomib therapy are associated with T cell immune dysfunctions, the use of immunomodulatory agents, such as thymosin, to enhance the immune function of T lymphocytes may be useful in preventing infections in these patients.
